# Supplementary figures and images for: Hospital length of stay throughout bed pathways and factors affecting this time: A non-concurrent cohort study of Colombia COVID-19 patients and an unCoVer network project
Source: PLoS One. 2023 Jul 26;18(7):e0278429. doi: 10.1371/journal.pone.0278429 (PMC10370719; doi:10.1371/journal.pone.0278429)

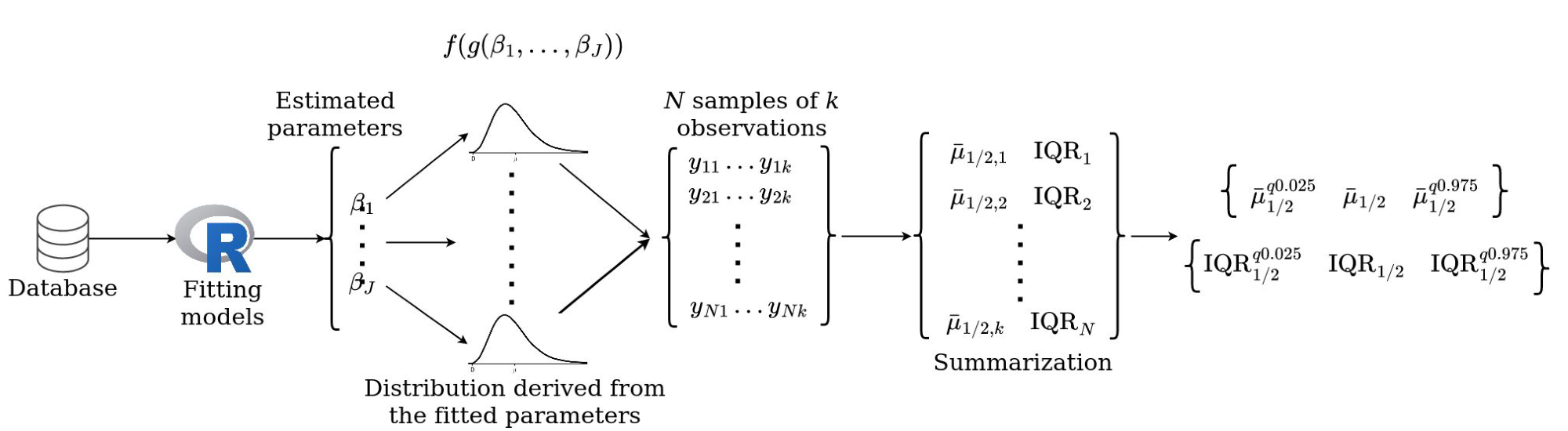

Supplement: S1 Fig — (TIF) [file pone.0278429.s001.tif]

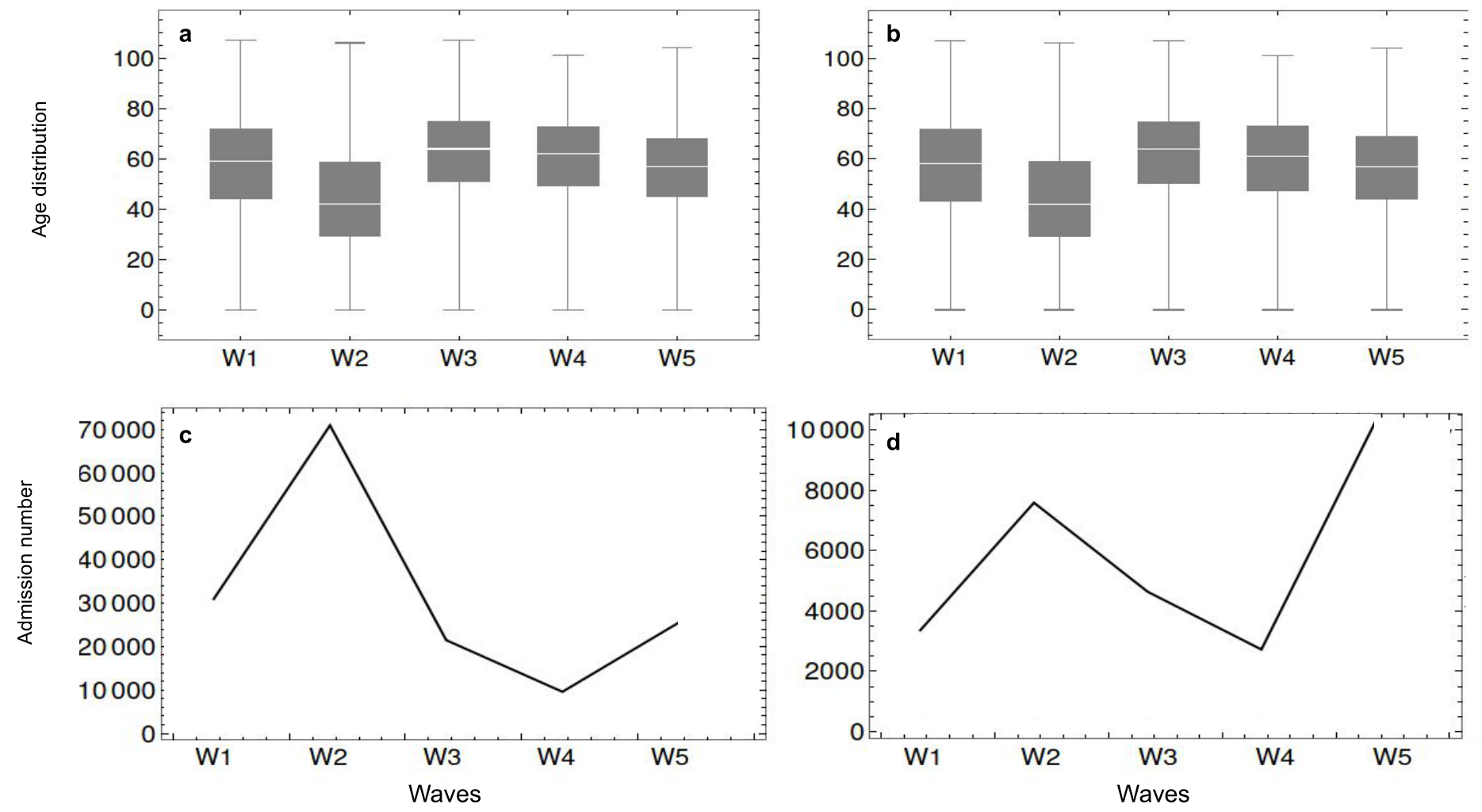

Supplement: S2 Fig — (a). The epidemic curve (black) is normalized with respect to the maximum number of cases. The first derivative curve (blue) is also normalized with respect to the maximum and minimum velocity value for positive and negative velocities, respectively. Gray line indicates the zero values. (b). The red line represents the amount of velocity points between the thresholds for each set of points (i.e., each daily velocity and three velocity points before and after each daily point). The velocity thresholds are 150 and -150, those are considered as low velocities of increment and decrement, respectively. The curve is also normalized by the maximum number of the counting. (c). The dashed lines in black indicate the start and the end of an epidemic wave. (d). Epidemic waves (gray bars are time windows not included). (TIF) [file pone.0278429.s002.tif]

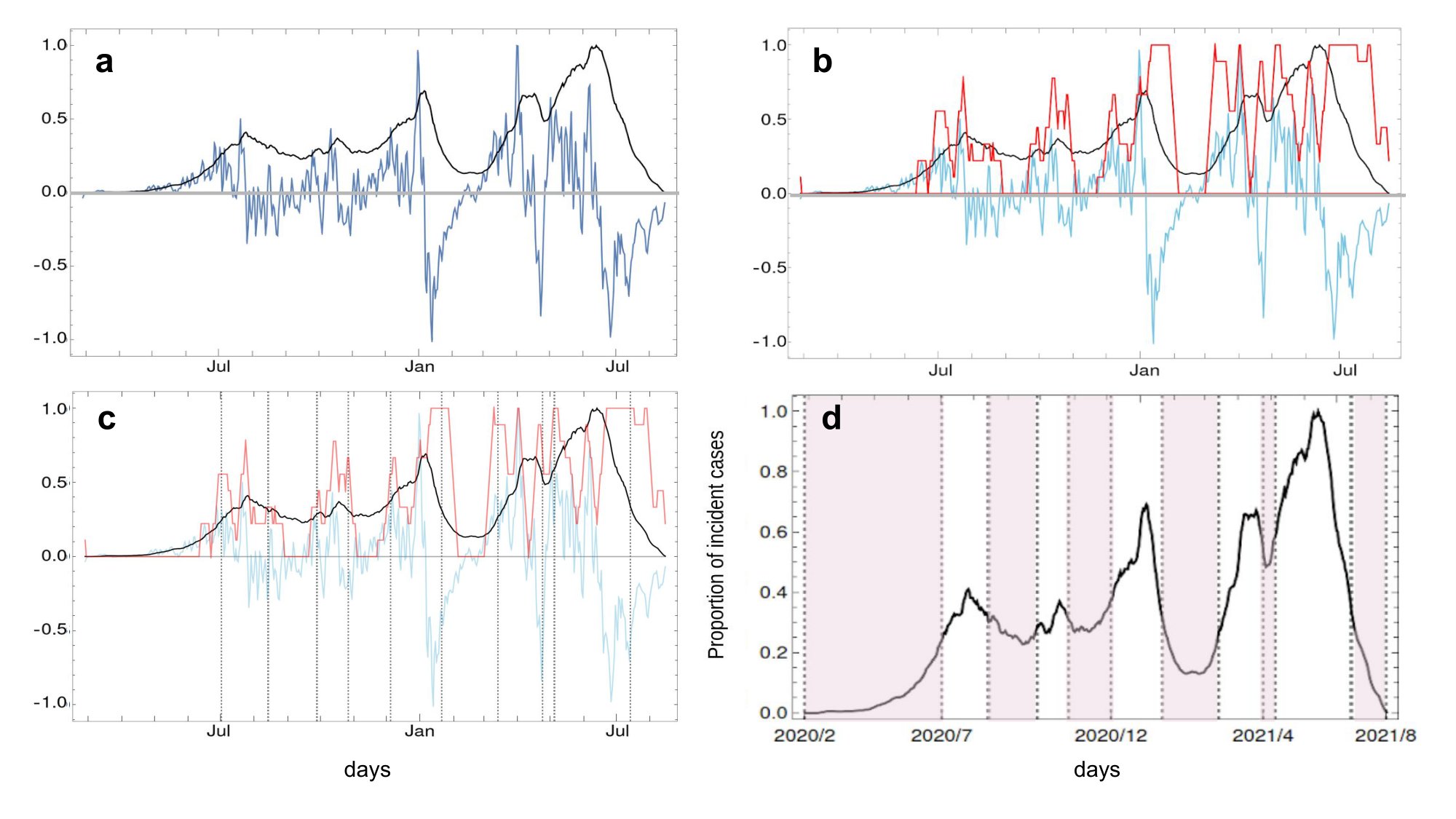

Supplement: S3 Fig — (a). The epidemic curve (black) is normalized with respect to the maximum number of cases. The first derivative curve (blue) is also normalized with respect to the maximum and minimum velocity value for positive and negative velocities, respectively. Gray line indicates the zero values. (b). The red line (the counting line) represents the amount of velocity points above or below the positive and negative threshold, respectively, for each set of points (i.e., each daily velocity and three velocity points before and after each daily point). The velocity thresholds are 200 and -200, those are considered as high velocities of increment and decrement, respectively. The curve is also normalized by the maximum number of the counting. (c). The dashed lines in black indicate the start and the end of a peak and valley. (d). Epidemic peaks (white) and valleys (pink). (TIF) [file pone.0278429.s003.tif]
