# Supplementary material for: Hospital length of stay throughout bed pathways and factors affecting this time: A non-concurrent cohort study of Colombia COVID-19 patients and an unCoVer network project
Source: PLoS One. 2023 Jul 26;18(7):e0278429. doi: 10.1371/journal.pone.0278429 (PMC10370719; doi:10.1371/journal.pone.0278429)
Supplement: S1 Table — Those were calculated in different AFT models for bed pathway, outcome, age, sex, waves, peaks and valleys, and vaccination period. (DOCX) [file pone.0278429.s005.docx]

### **S1 Table. Acceleration factors of the length of stay in each bed type and each bed pathway.** Those were calculated in different AFT models for bed pathway, outcome, age, sex, waves, peaks and valleys, and vaccination period.

| Covariable | **BP1** | **BP2** | **BP3** | | **Total BP3** |  | **BP4** | **Total BP4** |
| --- | --- | --- | --- | --- | --- | --- | --- | --- |
|  | **H** | **ICU** | **H** | **ICU** | **T** | **ICU** | **H** | **T** |
| **Bed Pathway BP3** | 0.95  (0.91-0.98) | 1.51  (1.46-1.56) | NA | NA | NA | NA | NA | NA |
| **BP4** | 0.76  (0.72-0.80) | 1.60  (1.52-1.68) | NA | NA | NA | NA | NA | NA |
| **Outcome**  **Recovery** | 1.94  (1.90-1.98) | 1.56  (1.51-1.61) | 1.16  (1.11-1.22) | 2.40  (2.28-2.52) | 1.88  (1.83-1.95) | 2.24  (1.89-2.66) | 1.15  (0.96-1.38)* | 2.15  (1.90-2.44) |
| **Age**  **26-50** | 1.44  (1.40-1.48) | 2.29  (2.12-2.46) | 0.96  (0.81-1.13)* | 1.07  (0.89-1.29)* | 1.03  (0.91-1.17)* | 1.20  (0.92-1.55)* | 0.68  (0.52-0.89) | 0.95  (0.79-1.16)* |
| **51-75** | 2.43  (2.36-2.49) | 3.34  (3.10-3.58) | 1.09  (0.93-1.28)* | 0.93  (0.78-1.12)* | 0.98  (0.87-1.11)* | 1.14  (0.89-1.47)* | 0.72  (0.55-0.94) | 0.923  (0.76-1.11)* |
| **>75** | 1.85  (1.80-1.91) | 2.23  (2.06-2.42) | 1.10  (0.93-1.30)* | 0.74  (0.62-0.90) | 0.86  (0.75-0.97) | 0.86  (0.65-1.15)* | 0.80  (0.59-1.08)* | 0.79  (0.63-0.97) |
| **Gender**  **Male** | 1.11  (1.09-1.13) | 1.22  (1.18-1.26) | 1.03  (0.98-1.08)* | 1.03  (0.97-1.08)* | 1.02  (0.99-1.06)* | 1.04  (0.95-1.14)* | 0.96  (0.87-1.06)* | 0.99  (0.93-1.07)* |
| **Waves**  **W2** | 0.18  (0.17-0.18) | 0.35  (0.33-0.37) | 0.58  (0.53-0.63) | 0.76  (0.69-0.83) | 0.67  (0.63-0.72) | 0.38  (0.31-0.46) | 1.31  (1.07-1.60) | 0.66  (0.57-0.77) |
| **W3** | 0.62  (0.60-0.63) | 1.38  (1.30-1.46) | 0.59  (0.55-0.64) | 1.02  (0.95-1.11)* | 0.79  (0.75-0.83) | 0.64  (0.52-0.78) | 1.08  (0.89-1.30)* | 0.70  (0.61-0.81) |
| **W4** | 0.46  (0.45-0.47) | 1.06  (0.99-1.13)* | 0.39  (0.35-0.43) | 0.69  (0.62-0.76) | 0.51  (0.48-0.55) | 0.44  (0.37-0.53) | 0.54  (0.45-0.64) | 0.43  (0.37-0.49) |
| **W5** | 0.81  (0.79-0.83) | 1.75  (1.67-1.84) | 0.43  (0.40-0.47) | 1.00  (0.92-1.09)* | 0.69  (0.65-0.73) | 0.62  (0.54-0.72) | 1.31  (1.14-1.51) | 1.01  (0.91-1.12)* |
| **Valleys/**  **Peaks**  **Valley** | 0.36  (0.36-0.37) | 0.38  (0.36-0.39) | 1.00  (0.95-1.08)* | 0.92  (0.86-0.99) | 0.94  (0.90-0.99) | 0.81  (0.72-0.91) | 0.64  (0.57-0.72) | 0.69  (0.64-0.75) |
| **Vaccination**  **Period**  **Yes** | 1.80  (1.77-1.84) | 2.16  (2.09-2.23) | 0.58  (0.55-0.61) | 0.90  (0.86-0.95) | 0.76  (0.73-0.79) | 1.11  (1.01-1.22) | 0.60  (0.55-0.66) | 1.03  (0.96-1.10)* |

GW: General Ward, ICU: Intensive Care Unit, T: Total, NA: Not applicable, *There is no significant difference with respect to the basal group. The basal groups are BP1 (for BPs when comparing the GW LoS), BP2 (for BPs when comparing the ICU LoS), Death (for outcome), <26 (for age), Women (for sex), W1 (for waves), Peaks (for Valleys and Peaks), No (for Vaccination period).
