# Supplementary material for: Hospital length of stay throughout bed pathways and factors affecting this time: A non-concurrent cohort study of Colombia COVID-19 patients and an unCoVer network project
Source: PLoS One. 2023 Jul 26;18(7):e0278429. doi: 10.1371/journal.pone.0278429 (PMC10370719; doi:10.1371/journal.pone.0278429)
Supplement: S2 Table — Those were calculated in different AFT models for bed pathway, outcome, age, sex, waves (W), peaks and valleys, and vaccination period. We get the lognormal distributions parameters from each AFT model and performance sampling to estimate the median and IQR. (DOCX) [file pone.0278429.s006.docx]

**S2 Table. Length of stay for GW (BP1) and ICU (BP2) pathways according to covariates.** Those were calculated in different AFT models for bed pathway, outcome, age, sex, waves (W), peaks and valleys, and vaccination period. We get the lognormal distributions parameters from each AFT model and performance sampling to estimate the median and IQR.

| **Covariable** | **BP1** | | | | **BP2** | | | |
| --- | --- | --- | --- | --- | --- | --- | --- | --- |
|  | **n** | **%** | **x** | **IQR** | **n** | **%** | **x** | **IQR** |
| **Bed Pathway BP1/2** | 184,340 | 94.38 | 6.49  (6.23-6.74) | 2.19 - 19.15 | 31,032 | 73.88 | 6.69  (6.48-6.93) | 2.65 - 16.88 |
| **BP3** | 7,632 | 3.91 | 6.16  (5.92-6.41) | 2.08 - 18.18 | 7,632 | 18.17 | 10.10  (9.78-10.44) | 4.00 - 25.47 |
| **BP4** | 3,341 | 1.71 | 4.92  (4.73-5.12) | 1.66 - 14.52 | 3,341 | 7.95 | 10.69  (10.33-11.05) | 4.24 - 26.95 |
| **Outcome**  **Death** | 32705 | 17.74 | 3.76  (3.61-3.91) | 1.27 - 11.12 | 13238 | 42.66 | 5.19  (5.01-5.37) | 2.00 - 13.44 |
| **Recovery** | 151635 | 82.26 | 7.30  (7.02-7.58) | 2.46 - 21.59 | 17794 | 57.34 | 8.09  (7.81-8.37) | 3.13 - 20.94 |
| **Age**  **1-25** | 18,024 | 9.93 | 3.57  (3.44-3.68) | 1.22 - 10.51 | 1,602 | 5.20 | 2.50  (2.41-2.56) | 0.89 - 12.12 |
| **26-50** | 62,173 | 34.27 | 5.16  (4.96-5.36) | 1.75 - 15.16 | 8,924 | 28.98 | 5.71  (5.51-5.85) | 2.60 - 35.25 |
| **51-75** | 75,003 | 41.34 | 8.68  (8.34-9.03) | 2.95 - 25.52 | 16,294 | 52.92 | 8.33  (8.05-8.54) | 4.06 - 55.21 |
| **>75** | 26,211 | 14.45 | 6.63  (6.38-6.90) | 2.26 - 19.49 | 3,970 | 12.89 | 5.58  (5.39-5.72) | 2.58 - 35.06 |
| **Gender**  **Women** | 84170 | 45.66 | 6.12  (5.88-6.37) | 2.04 - 18.36 | 12437 | 40.08 | 5.95  (5.74-6.16) | 2.28 - 15.54 |
| **Men** | 100170 | 54.34 | 6.81  (6.54-7.09) | 2.27 - 20.41 | 18595 | 59.92 | 7.24  (6.99-7.50) | 2.77 - 18.92 |
| **Waves**  **W1** | 31158 | 19.55 | 12.07  (11.69-12.47) | 4.99 - 29.24 | 3140 | 11.46 | 5.71  (5.54-5.88) | 2.50 - 13.03 |
| **W2** | 71044 | 44.57 | 2.14  (2.07-2.21) | 0.88 - 5.17 | 7346 | 26.81 | 2.00  (1.95-2.07) | 0.88 - 4.58 |
| **W3** | 21681 | 13.60 | 7.45  (7.20-7.69) | 3.07 - 18.03 | 4410 | 16.09 | 7.89  (7.65-8.13) | 3.45 - 17.99 |
| **W4** | 9812 | 6.15 | 5.57  (5.39-5.75) | 2.30 - 13.48 | 2507 | 9.15 | 6.07  (5.89-6.26) | 2.66 - 13.85 |
| **W5** | 25701 | 16.12 | 9.80  (9.49-10.12) | 4.05 - 23.73 | 9994 | 36.48 | 10.01  (9.71-10.32) | 4.39 - 22.84 |
| **Peaks** | 40,632 | 33.78 | 5.17  (5.04-5.31) | 2.53 - 10.59 | 10,787 | 49.55 | 6.24  (6.07-6.42) | 2.93 - 13.27 |
| **Valleys** | 79,636 | 66.21 | 1.89  (1.84-1.94) | 0.92 - 3.86 | 10,981 | 50.44 | 2.35  (2.28-2.42) | 1.10 - 5.00 |
| **Vaccination**  **Period**  **No** | 143,589 | 77.89 | 5.69  (5.47-5.92) | 1.92 - 16.86 | 16,744 | 53.96 | 4.69  (4.54-4.85) | 1.86 - 11.85 |
| **Yes** | 40,751 | 22.10 | 10.27  (9.87-10.68) | 3.47 - 30.42 | 14,288 | 46.04 | 10.16  (9.82-10.51) | 4.02 - 25.66 |

GW General Ward bed, ICU Intensive Care Unit, n number of hospital admissions entering each bed pathway , % proportion of hospital admissions entering each bed pathway, $x$ Median and 95% C.I., IQR Interquartile range.
