# Supplementary material for: Hospital length of stay throughout bed pathways and factors affecting this time: A non-concurrent cohort study of Colombia COVID-19 patients and an unCoVer network project
Source: PLoS One. 2023 Jul 26;18(7):e0278429. doi: 10.1371/journal.pone.0278429 (PMC10370719; doi:10.1371/journal.pone.0278429)
Supplement: S3 Table — We get the Lognormal distributions parameters from each AFT model and performance sampling to estimate the median and IQR. (DOCX) [file pone.0278429.s007.docx]

### **S3 Table. Median and IQR of the length of stay in GW (BP1) and ICU (BP2).** We get the Lognormal distributions parameters from each AFT model and performance sampling to estimate the median and IQR.

| **Geographic region** | **BP1** | | | | **BP2** | | | |
| --- | --- | --- | --- | --- | --- | --- | --- | --- |
|  | **n** | **%** | **x** | **IQR** | **n** | **%** | **x** | **IQR** |
| **Amazonas** | 269 | 0.14 | 4.74  (4.56-4.93) | 1.60 - 14.08 | 56 | 0.18 | 8.38  (8.10-8.68) | 3.28 - 14.08 |
| **Antioquia** | 20,663 | 11.20 | 4.92  (4.72-5.12) | 1.65 - 14.60 | 2,974 | 9.58 | 4.98  (4.81-5.15) | 1.95 - 14.60 |
| **Arauca** | 586 | 0.31 | 4.52  (4.35-4.70) | 1.52 - 13.43 | 70 | 0.22 | 3.76  (3.64-3.89) | 1.47 - 13.42 |
| **Atlantico** | 4,483 | 2.43 | 6.80  (6.54-7.08) | 2.29 - 20.20 | 881 | 2.84 | 5.39  (5.21-5.58) | 2.11 - 20.19 |
| **Barranquilla** | 8,063 | 4.37 | 6.22  (5.98-6.47) | 2.09 - 18.47 | 1,835 | 5.91 | 5.59  (5.41-5.78) | 2.19 - 18.47 |
| **Bogota** | 57,003 | 30.92 | 7.46  (7.17-7.75) | 2.51 - 22.13 | 12,870 | 41.47 | 9.41  (9.09-9.74) | 3.68 - 22.13 |
| **Bolivar** | 1,211 | 0.65 | 7.95  (7.65-8.27) | 2.68 - 23.61 | 121 | 0.39 | 5.30  (5.12-5.48) | 2.07 - 23.61 |
| **Boyaca** | 3,074 | 1.66 | 5.16  (4.96-5.38) | 1.74 - 15.33 | 371 | 1.19 | 6.03  (5.83-6.23) | 2.36 - 15.33 |
| **Caldas** | 2,992 | 1.62 | 4.71  (4.53-4.91) | 1.59 - 14.00 | 334 | 1.08 | 5.27  (5.09-5.45) | 2.06 - 14.00 |
| **Caqueta** | 1,558 | 0.84 | 6.79  (6.537.06) | 2.28 - 20.15 | 186 | 0.60 | 4.26  (4.12-4.41) | 1.67 - 20.15 |
| **Cartagena** | 2,436 | 1.32 | 4.17  (4.01-4.34) | 1.40 - 12.38 | 406 | 1.30 | 4.23  (4.09-4.38) | 1.65 - 12.38 |
| **Casanare** | 1,091 | 0.59 | 8.54  (8.21-8.88) | 2.87 - 25.34 | 178 | 0.57 | 6.13  (5.92-6.34) | 2.40 - 25.35 |
| **Cauca** | 2,165 | 1.17 | 6.48  (6.22-6.73) | 2.18 - 19.23 | 380 | 1.22 | 8.34  (8.06-8.64) | 3.27 - 19.23 |
| **Cesar** | 4,515 | 2.44 | 8.74  (8.41-9.09) | 2.94 - 25.95 | 502 | 1.62 | 5.43  (5.25-5.62) | 2.13 - 25.95 |
| **Choco** | 695 | 0.38 | 9.60  (9.22-9.97) | 3.23 - 28.49 | 89 | 0.29 | 8.10  (7.83-8.38) | 3.17 - 28.49 |
| **Cordoba** | 4,265 | 2.31 | 6.85  (6.58-7.12) | 2.30 - 20.33 | 553 | 1.78 | 4.49  (4.34-4.64) | 1.76 - 20.33 |
| **Cundinamarca** | 9,066 | 4.2 | 8.23  (7.90-8.56) | 2.77 - 24.43 | 942 | 3.03 | 4.71  (4.55-4.87) | 1.84 - 24.43 |
| **Guainia** | 83 | 0.04 | 2.24  (2.15-2.33) | 0.75- 6.66 | 16 | 0.05 | 2.19  (2.12-2.27) | 0.86- 6.66 |
| **Guajira** | 1,516 | 0.82 | 7.64  (7.34-7.95) | 2.57 - 22.68 | 201 | 0.64 | 4.44  (4.30-4.60) | 1.74 - 22.68 |
| **Guaviare** | 186 | 0.10 | 3.02  (2.90-3.14) | 1.01 - 8.96 | 17 | 0.05 | 2.26  (2.19-2.34) | 0.88 - 8.96 |
| **Huila** | 4,949 | 2.68 | 4.48  (4.31-4.66) | 1.51 - 13.30 | 839 | 2.70 | 5.08  (4.91-5.26) | 1.99 - 13.30 |
| **Magdalena** | 1,438 | 0.78 | 8.62  (8.28-8.97) | 2.90 - 25.59 | 194 | 0.62 | 4.53  (4.38-4.69) | 1.77 - 25.59 |
| **Meta** | 2,516 | 1.36 | 3.87  (3.71-4.03) | 1.30 - 11.47 | 287 | 0.92 | 3.06  (2.96-3.17) | 1.20 - 11.48 |
| **Nariño** | 4,182 | 2.27 | 8.23  (7.90-8.56) | 2.77- 24.42 | 531 | 1.71 | 6.63  (6.40-6.86) | 2.59 - 24.42 |
| **Norte de Santander** | 6,879 | 3.73 | 5.85  (5.63-6.08) | 1.97 - 17.38 | 1,428 | 4.60 | 6.47  (6.26-6.70) | 2.53 - 17.38 |
| **Putumayo** | 1,263 | 0.68 | 14.96  (14.37-15.57) | 5.04 - 44.40 | 110 | 0.35 | 8.90  (8.60-9.20) | 3.48 - 44.40 |
| **Quindio** | 1,865 | 1.01 | 3.38  (3.24-3.51) | 1.14 - 10.03 | 162 | 0.52 | 2.59  (2.51-2.68) | 1.01 - 10.03 |
| **Risaralda** | 2,812 | 1.52 | 4.94  (4.75-5.15) | 1.66 - 14.68 | 364 | 1.17 | 4.97  (4.81-5.14) | 1.95 - 14.68 |
| **San Andres** | 240 | 0.13 | 7.45  (7.16-7.75) | 2.51 - 22.13 | 37 | 0.12 | 4.62  (4.46-4.79) | 1.81 - 22.13 |
| **Santander** | 9,111 | 4.94 | 6.63  (6.37-6.89) | 2.23- 19.67 | 839 | 2.70 | 4.55  (4.40-4.71) | 1.78 - 19.67 |
| **Santa Marta** | 1,986 | 1.07 | 6.95  (6.69-7.24) | 2.34 - 20.65 | 334 | 1.07 | 5.31  (5.13-5.50) | 2.08 - 20.65 |
| **Sucre** | 2,311 | 1.25 | 7.88  (7.57-8.19) | 2.65 - 23.39 | 278 | 0.89 | 3.52  (3.40-3.64) | 1.38 - 23.39 |
| **Tolima** | 4,923 | 2.67 | 6.15  (5.92-6.40) | 2.07 - 18.27 | 510 | 1.64 | 4.62  (4.47-4.78) | 1.81 - 18.27 |
| **Valle** | 13,795 | 7.48 | 6.08  (5.84-6.33) | 2.05 - 18.07 | 2,113 | 6.80 | 6.00  (5.80-6.20) | 2.35 - 18.07 |
| **Vaupes** | 78 | 0.04 | 4.54  (4.36-4.72) | 1.53 - 13.48 | 14 | 0.04 | 2.21  (2.14-2.29) | 0.87 - 12.92 |
| **Vichada** | 72 | 0.039 | 5.57  (5.35-5.78) | 1.87 - 16.52 | 10 | 0.03 | 5.52  (5.34-5.71) | 2.16 - 16.52 |
| **Total** | 184340 |  |  |  | 31032 |  |  |  |

GW General Ward bed, ICU Intensive Care Unit, n number of hospital admissions entering each bed pathway , % proportion of hospital admissions entering each bed pathway, $x$ Median and 95% C.I., IQR Interquartile range.
