# Supplementary material for: Hospital length of stay throughout bed pathways and factors affecting this time: A non-concurrent cohort study of Colombia COVID-19 patients and an unCoVer network project
Source: PLoS One. 2023 Jul 26;18(7):e0278429. doi: 10.1371/journal.pone.0278429 (PMC10370719; doi:10.1371/journal.pone.0278429)
Supplement: S1 File — (DOCX) [file pone.0278429.s009.docx]

**SUPPLEMENTARY INFORMATION: An UnCover analysis of bed pathways and hospital Length of Stay (LoS): A retrospective cohort study of Colombian COVID-19 patients**

**Table of content:**

[**Methodology 2**](#_ubedezhnmnmx)

[**Estimating the median, IQR, and confidence intervals 2**](#_be43bkb8jdaz)

[**S1 Fig. Estimation of the median, IQR, and their 95% confidence interval of each AFT model. 2**](#_j4koggqp2p3y)

[**Estimating the waves 2**](#_nfbi0mqyx1j4)

[**S2 Fig. Estimation of waves of the epidemic curve of SARS-CoV-2 daily infected cases in Colombia. 3**](#_s8hg55rchtv2)

[**Estimating the peaks and valleys 3**](#_hdq0912h40gs)

[**S3 Fig. Estimation of peaks and valleys of the epidemic curve of SARS-CoV-2 daily infected cases in Colombia. 4**](#_34olpna9896r)

[**Results 4**](#_30i0bzxycgvx)

[**S1 Table. Acceleration factors of the length of stay in each bed type and each bed pathway. 4**](#_xz9hjvb4n219)

[**S2 Table. Length of stay for GW (BP1) and ICU (BP2) pathways according to covariates. 5**](#_w0ycur7w9xrb)

[**S3 Table. Median and IQR of the length of stay in GW (BP1) and ICU (BP2). 6**](#_t3zdwtft3d45)

[**S4 Fig. Age distribution and admission number throughout the waves. 9**](#_cwo2ymtlh6xc)

# Methodology

## Estimating the median, IQR, and confidence intervals

**
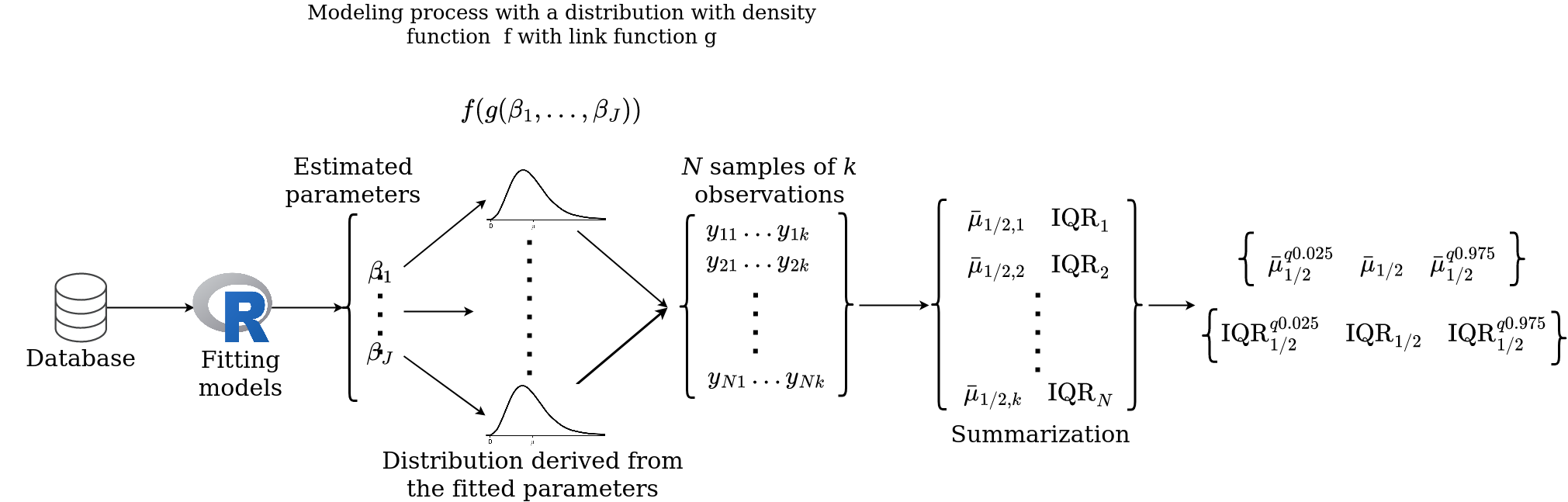
**

### S1 Fig. Estimation of the median, IQR, and their 95% confidence interval of each AFT model.

#

## Estimating the waves

We followed the next steps to identify the waves in the epidemic curve of Colombia SARS-CoV-2 infected cases. First, we smoothed the epidemic curve as in [(1)](https://paperpile.com/c/iYjjEL/CyLT):

$\underline{x}_{k}$ = $\frac{1}{7}\sum_{j = i-3}^{j=i+3} x_{j}$

Where $\underline{x}_{k}$ is the mean for each daily incidence $k$, this estimation includes each day $i$ and three days after $j= i-3$ and before each day $j=i+3$. Second, we calculated the first derivate (i.e., velocity or change of the daily incidence of cases) as in [(1)](https://paperpile.com/c/iYjjEL/CyLT):

$\frac{d \underline{x}}{d t}\approx\frac{1}{2}(\underline{x}_{k+1}-\underline{x}_{k-1})$

The epidemic curve has peaks and valleys, both of them are characterized by low velocities or zero velocities (Figure S1a). The velocity remains oscillating with low values around zero for a certain time window depending on the peak or valley size. This time window is longer in valleys than in peaks, which helps to delimit the waves. A wave is a time window that includes the increment, peak, and decrement in the number of daily cases, and it is found between valleys.

**
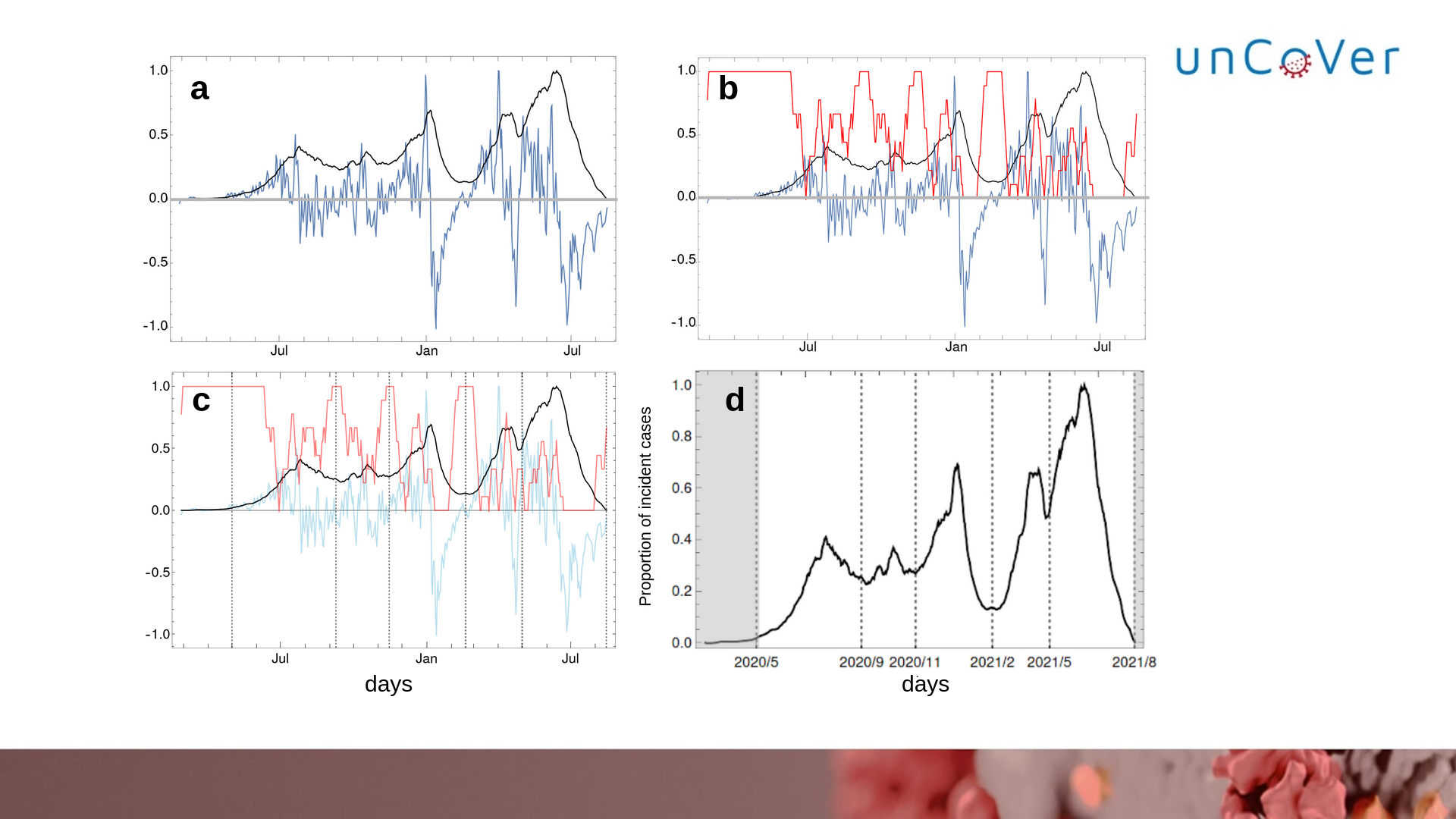
**

### S2 Fig. Estimation of waves of the epidemic curve of SARS-CoV-2 daily infected cases in Colombia.

(**a**). The epidemic curve (black) is normalized with respect to the maximum number of cases. The first derivative curve (blue) is also normalized with respect to the maximum and minimum velocity value for positive and negative velocities, respectively. Gray line indicates the zero values. (**b**). The red line represents the amount of velocity points between the thresholds for each set of points (i.e., each daily velocity and three velocity points before and after each daily point). The velocity thresholds are 150 and -150, those are considered as low velocities of increment and decrement, respectively. The curve is also normalized by the maximum number of the counting. (**c**). The dashed lines in black indicate the start and the end of an epidemic wave. (**d**). Epidemic waves (gray bars are time windows not included).

Third, we chose a positive and negative threshold of velocity. A particular velocity value, from the first derivative curve, is considered low when it is between these thresholds. Fourth, we evaluated if each daily velocity together with some neighbor velocities are between these thresholds. The neighbor velocities could be one or more than one velocity point after and before each daily point. Then, we counted the amount of velocity points between the thresholds for each set of velocities constituted by each daily velocity and its neighbors (Figure S1b). Finally, we identified the valleys as the zones of maximum counting for low velocities and manually delimited the start and the end of a wave as is shown in Figure S1c-d.

## Estimating the peaks and valleys

To identify the peaks and valleys, we also followed the first two steps previously described for waves. Contrary to waves, the peaks and valleys are delimited by zones with the highest values in velocities, in other words, they are between zones of high velocity. As a consequence, the third step was to choose positive and negative thresholds above and below which the velocity values are considered as high, respectively. Then, we also chose consecutive sets (i.e., each daily velocity together with some neighbor velocities) of daily velocity points to classify their velocities. We counted the amount of velocity points above and below the positive and negative threshold, respectively. As a result, we also had a line of the counting for each daily velocity set (Figure S2b). Finally, we delimited the beginning and the end of each peak and valley according to the line of counting and the epidemic curve (Figure S2c-d).


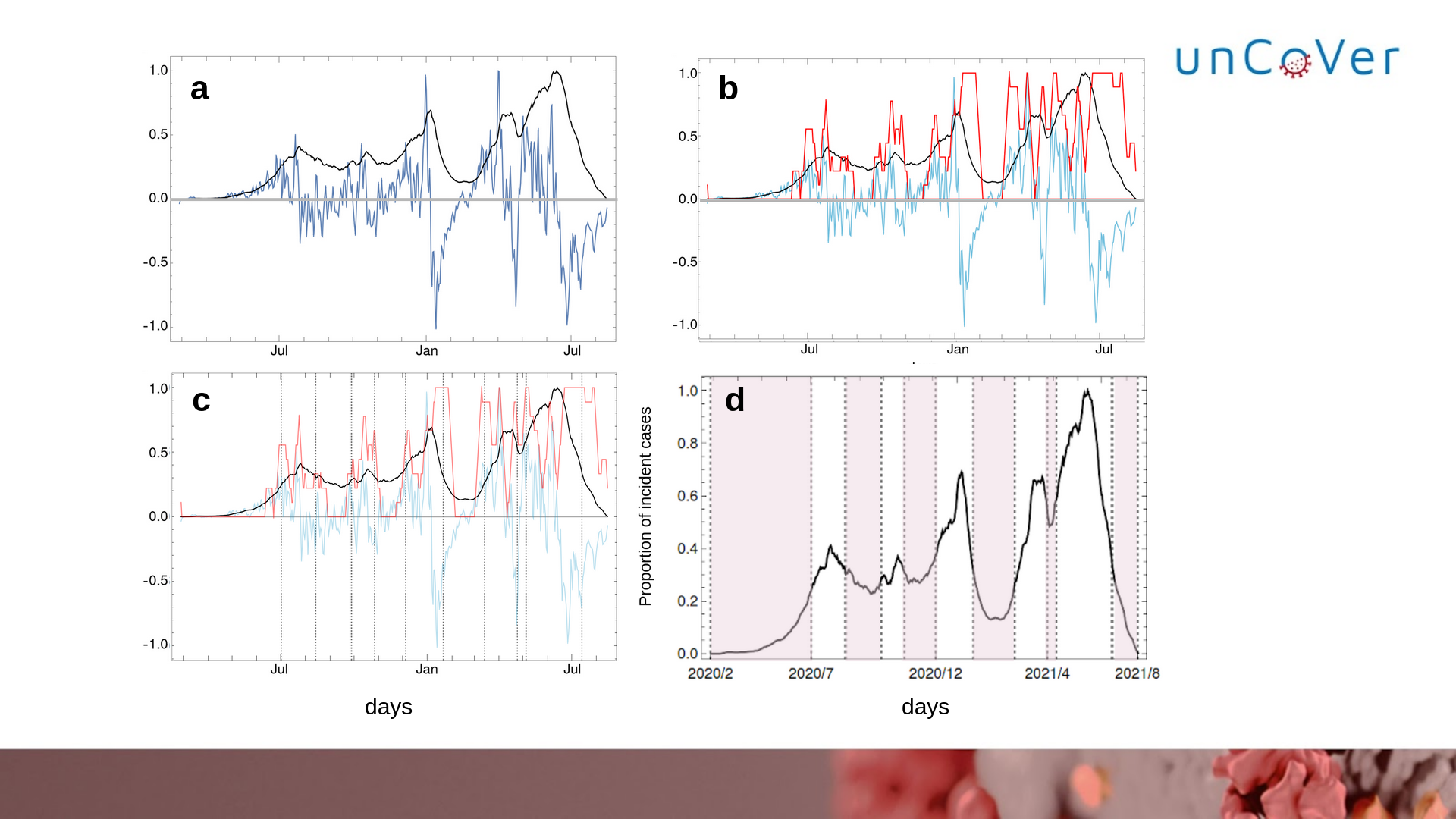


### S3 Fig. Estimation of peaks and valleys of the epidemic curve of SARS-CoV-2 daily infected cases in Colombia.

(**a**). The epidemic curve (black) is normalized with respect to the maximum number of cases. The first derivative curve (blue) is also normalized with respect to the maximum and minimum velocity value for positive and negative velocities, respectively. Gray line indicates the zero values. (**b**). The red line (the counting line) represents the amount of velocity points above or below the positive and negative threshold, respectively, for each set of points (i.e., each daily velocity and three velocity points before and after each daily point). The velocity thresholds are 200 and -200, those are considered as high velocities of increment and decrement, respectively. The curve is also normalized by the maximum number of the counting. (**c**). The dashed lines in black indicate the start and the end of a peak and valley. (**d**). Epidemic peaks (white) and valleys (pink).

# Results

### **S1 Table.** Acceleration factors of the length of stay in each bed type and each bed pathway. Those were calculated in different AFT models for bed pathway, outcome, age, sex, waves, peaks and valleys, and vaccination period.

| Covariable | **BP1** | **BP2** | **BP3** | | **Total BP3** |  | **BP4** | **Total BP4** |
| --- | --- | --- | --- | --- | --- | --- | --- | --- |
|  | **H** | **ICU** | **H** | **ICU** | **T** | **ICU** | **H** | **T** |
| **Bed Pathway BP3** | 0.95  (0.91-0.98) | 1.51  (1.46-1.56) | NA | NA | NA | NA | NA | NA |
| **BP4** | 0.76  (0.72-0.80) | 1.60  (1.52-1.68) | NA | NA | NA | NA | NA | NA |
| **Outcome**  **Recovery** | 1.94  (1.90-1.98) | 1.56  (1.51-1.61) | 1.16  (1.11-1.22) | 2.40  (2.28-2.52) | 1.88  (1.83-1.95) | 2.24  (1.89-2.66) | 1.15  (0.96-1.38)* | 2.15  (1.90-2.44) |
| **Age**  **26-50** | 1.44  (1.40-1.48) | 2.29  (2.12-2.46) | 0.96  (0.81-1.13)* | 1.07  (0.89-1.29)* | 1.03  (0.91-1.17)* | 1.20  (0.92-1.55)* | 0.68  (0.52-0.89) | 0.95  (0.79-1.16)* |
| **51-75** | 2.43  (2.36-2.49) | 3.34  (3.10-3.58) | 1.09  (0.93-1.28)* | 0.93  (0.78-1.12)* | 0.98  (0.87-1.11)* | 1.14  (0.89-1.47)* | 0.72  (0.55-0.94) | 0.923  (0.76-1.11)* |
| **>75** | 1.85  (1.80-1.91) | 2.23  (2.06-2.42) | 1.10  (0.93-1.30)* | 0.74  (0.62-0.90) | 0.86  (0.75-0.97) | 0.86  (0.65-1.15)* | 0.80  (0.59-1.08)* | 0.79  (0.63-0.97) |
| **Gender**  **Male** | 1.11  (1.09-1.13) | 1.22  (1.18-1.26) | 1.03  (0.98-1.08)* | 1.03  (0.97-1.08)* | 1.02  (0.99-1.06)* | 1.04  (0.95-1.14)* | 0.96  (0.87-1.06)* | 0.99  (0.93-1.07)* |
| **Waves**  **W2** | 0.18  (0.17-0.18) | 0.35  (0.33-0.37) | 0.58  (0.53-0.63) | 0.76  (0.69-0.83) | 0.67  (0.63-0.72) | 0.38  (0.31-0.46) | 1.31  (1.07-1.60) | 0.66  (0.57-0.77) |
| **W3** | 0.62  (0.60-0.63) | 1.38  (1.30-1.46) | 0.59  (0.55-0.64) | 1.02  (0.95-1.11)* | 0.79  (0.75-0.83) | 0.64  (0.52-0.78) | 1.08  (0.89-1.30)* | 0.70  (0.61-0.81) |
| **W4** | 0.46  (0.45-0.47) | 1.06  (0.99-1.13)* | 0.39  (0.35-0.43) | 0.69  (0.62-0.76) | 0.51  (0.48-0.55) | 0.44  (0.37-0.53) | 0.54  (0.45-0.64) | 0.43  (0.37-0.49) |
| **W5** | 0.81  (0.79-0.83) | 1.75  (1.67-1.84) | 0.43  (0.40-0.47) | 1.00  (0.92-1.09)* | 0.69  (0.65-0.73) | 0.62  (0.54-0.72) | 1.31  (1.14-1.51) | 1.01  (0.91-1.12)* |
| **Valleys/**  **Peaks**  **Valley** | 0.36  (0.36-0.37) | 0.38  (0.36-0.39) | 1.00  (0.95-1.08)* | 0.92  (0.86-0.99) | 0.94  (0.90-0.99) | 0.81  (0.72-0.91) | 0.64  (0.57-0.72) | 0.69  (0.64-0.75) |
| **Vaccination**  **Period**  **Yes** | 1.80  (1.77-1.84) | 2.16  (2.09-2.23) | 0.58  (0.55-0.61) | 0.90  (0.86-0.95) | 0.76  (0.73-0.79) | 1.11  (1.01-1.22) | 0.60  (0.55-0.66) | 1.03  (0.96-1.10)* |

GW: General Ward, ICU: Intensive Care Unit, T: Total, NA: Not applicable, *There is no significant difference with respect to the basal group. The basal groups are BP1 (for BPs when comparing the GW LoS), BP2 (for BPs when comparing the ICU LoS), Death (for outcome), <26 (for age), Women (for sex), W1 (for waves), Peaks (for Valleys and Peaks), No (for Vaccination period).

#

### S2 Table. Length of stay for GW (BP1) and ICU (BP2) pathways according to covariates.

Those were calculated in different AFT models for bed pathway, outcome, age, sex, waves (W), peaks and valleys, and vaccination period. We get the lognormal distributions parameters from each AFT model and performance sampling to estimate the median and IQR.

| **Covariable** | **BP1** | | | | **BP2** | | | |
| --- | --- | --- | --- | --- | --- | --- | --- | --- |
|  | **n** | **%** | **x** | **IQR** | **n** | **%** | **x** | **IQR** |
| **Bed Pathway BP1/2** | 184,340 | 94.38 | 6.49  (6.23-6.74) | 2.19 - 19.15 | 31,032 | 73.88 | 6.69  (6.48-6.93) | 2.65 - 16.88 |
| **BP3** | 7,632 | 3.91 | 6.16  (5.92-6.41) | 2.08 - 18.18 | 7,632 | 18.17 | 10.10  (9.78-10.44) | 4.00 - 25.47 |
| **BP4** | 3,341 | 1.71 | 4.92  (4.73-5.12) | 1.66 - 14.52 | 3,341 | 7.95 | 10.69  (10.33-11.05) | 4.24 - 26.95 |
| **Outcome**  **Death** | 32705 | 17.74 | 3.76  (3.61-3.91) | 1.27 - 11.12 | 13238 | 42.66 | 5.19  (5.01-5.37) | 2.00 - 13.44 |
| **Recovery** | 151635 | 82.26 | 7.30  (7.02-7.58) | 2.46 - 21.59 | 17794 | 57.34 | 8.09  (7.81-8.37) | 3.13 - 20.94 |
| **Age**  **1-25** | 18,024 | 9.93 | 3.57  (3.44-3.68) | 1.22 - 10.51 | 1,602 | 5.20 | 2.50  (2.41-2.56) | 0.89 - 12.12 |
| **26-50** | 62,173 | 34.27 | 5.16  (4.96-5.36) | 1.75 - 15.16 | 8,924 | 28.98 | 5.71  (5.51-5.85) | 2.60 - 35.25 |
| **51-75** | 75,003 | 41.34 | 8.68  (8.34-9.03) | 2.95 - 25.52 | 16,294 | 52.92 | 8.33  (8.05-8.54) | 4.06 - 55.21 |
| **>75** | 26,211 | 14.45 | 6.63  (6.38-6.90) | 2.26 - 19.49 | 3,970 | 12.89 | 5.58  (5.39-5.72) | 2.58 - 35.06 |
| **Gender**  **Women** | 84170 | 45.66 | 6.12  (5.88-6.37) | 2.04 - 18.36 | 12437 | 40.08 | 5.95  (5.74-6.16) | 2.28 - 15.54 |
| **Men** | 100170 | 54.34 | 6.81  (6.54-7.09) | 2.27 - 20.41 | 18595 | 59.92 | 7.24  (6.99-7.50) | 2.77 - 18.92 |
| **Waves**  **W1** | 31158 | 19.55 | 12.07  (11.69-12.47) | 4.99 - 29.24 | 3140 | 11.46 | 5.71  (5.54-5.88) | 2.50 - 13.03 |
| **W2** | 71044 | 44.57 | 2.14  (2.07-2.21) | 0.88 - 5.17 | 7346 | 26.81 | 2.00  (1.95-2.07) | 0.88 - 4.58 |
| **W3** | 21681 | 13.60 | 7.45  (7.20-7.69) | 3.07 - 18.03 | 4410 | 16.09 | 7.89  (7.65-8.13) | 3.45 - 17.99 |
| **W4** | 9812 | 6.15 | 5.57  (5.39-5.75) | 2.30 - 13.48 | 2507 | 9.15 | 6.07  (5.89-6.26) | 2.66 - 13.85 |
| **W5** | 25701 | 16.12 | 9.80  (9.49-10.12) | 4.05 - 23.73 | 9994 | 36.48 | 10.01  (9.71-10.32) | 4.39 - 22.84 |
| **Peaks** | 40,632 | 33.78 | 5.17  (5.04-5.31) | 2.53 - 10.59 | 10,787 | 49.55 | 6.24  (6.07-6.42) | 2.93 - 13.27 |
| **Valleys** | 79,636 | 66.21 | 1.89  (1.84-1.94) | 0.92 - 3.86 | 10,981 | 50.44 | 2.35  (2.28-2.42) | 1.10 - 5.00 |
| **Vaccination**  **Period**  **No** | 143,589 | **77.89** | 5.69  (5.47-5.92) | 1.92 - 16.86 | 16,744 | 53.96 | 4.69  (4.54-4.85) | 1.86 - 11.85 |
| **Yes** | 40,751 | **22.10** | 10.27  (9.87-10.68) | 3.47 - 30.42 | 14,288 | 46.04 | 10.16  (9.82-10.51) | 4.02 - 25.66 |

GW General Ward bed, ICU Intensive Care Unit, n number of hospital admissions entering each bed pathway , % proportion of hospital admissions entering each bed pathway, $x$ Median and 95% C.I., IQR Interquartile range.

### S3 Table. Median and IQR of the length of stay in GW (BP1) and ICU (BP2).

We get the Lognormal distributions parameters from each AFT model and performance sampling to estimate the median and IQR.

| **Geographic region** | **BP1** | | | | **BP2** | | | |
| --- | --- | --- | --- | --- | --- | --- | --- | --- |
|  | **n** | **%** | **x** | **IQR** | **n** | **%** | **x** | **IQR** |
| **Amazonas** | 269 | 0.14 | 4.74  (4.56-4.93) | 1.60 - 14.08 | 56 | 0.18 | 8.38  (8.10-8.68) | 3.28 - 14.08 |
| **Antioquia** | 20,663 | 11.20 | 4.92  (4.72-5.12) | 1.65 - 14.60 | 2,974 | 9.58 | 4.98  (4.81-5.15) | 1.95 - 14.60 |
| **Arauca** | 586 | 0.31 | 4.52  (4.35-4.70) | 1.52 - 13.43 | 70 | 0.22 | 3.76  (3.64-3.89) | 1.47 - 13.42 |
| **Atlantico** | 4,483 | 2.43 | 6.80  (6.54-7.08) | 2.29 - 20.20 | 881 | 2.84 | 5.39  (5.21-5.58) | 2.11 - 20.19 |
| **Barranquilla** | 8,063 | 4.37 | 6.22  (5.98-6.47) | 2.09 - 18.47 | 1,835 | 5.91 | 5.59  (5.41-5.78) | 2.19 - 18.47 |
| **Bogota** | 57,003 | 30.92 | 7.46  (7.17-7.75) | 2.51 - 22.13 | 12,870 | 41.47 | 9.41  (9.09-9.74) | 3.68 - 22.13 |
| **Bolivar** | 1,211 | 0.65 | 7.95  (7.65-8.27) | 2.68 - 23.61 | 121 | 0.39 | 5.30  (5.12-5.48) | 2.07 - 23.61 |
| **Boyaca** | 3,074 | 1.66 | 5.16  (4.96-5.38) | 1.74 - 15.33 | 371 | 1.19 | 6.03  (5.83-6.23) | 2.36 - 15.33 |
| **Caldas** | 2,992 | 1.62 | 4.71  (4.53-4.91) | 1.59 - 14.00 | 334 | 1.08 | 5.27  (5.09-5.45) | 2.06 - 14.00 |
| **Caqueta** | 1,558 | 0.84 | 6.79  (6.537.06) | 2.28 - 20.15 | 186 | 0.60 | 4.26  (4.12-4.41) | 1.67 - 20.15 |
| **Cartagena** | 2,436 | 1.32 | 4.17  (4.01-4.34) | 1.40 - 12.38 | 406 | 1.30 | 4.23  (4.09-4.38) | 1.65 - 12.38 |
| **Casanare** | 1,091 | 0.59 | 8.54  (8.21-8.88) | 2.87 - 25.34 | 178 | 0.57 | 6.13  (5.92-6.34) | 2.40 - 25.35 |
| **Cauca** | 2,165 | 1.17 | 6.48  (6.22-6.73) | 2.18 - 19.23 | 380 | 1.22 | 8.34  (8.06-8.64) | 3.27 - 19.23 |
| **Cesar** | 4,515 | 2.44 | 8.74  (8.41-9.09) | 2.94 - 25.95 | 502 | 1.62 | 5.43  (5.25-5.62) | 2.13 - 25.95 |
| **Choco** | 695 | 0.38 | 9.60  (9.22-9.97) | 3.23 - 28.49 | 89 | 0.29 | 8.10  (7.83-8.38) | 3.17 - 28.49 |
| **Cordoba** | 4,265 | 2.31 | 6.85  (6.58-7.12) | 2.30 - 20.33 | 553 | 1.78 | 4.49  (4.34-4.64) | 1.76 - 20.33 |
| **Cundinamarca** | 9,066 | 4.2 | 8.23  (7.90-8.56) | 2.77 - 24.43 | 942 | 3.03 | 4.71  (4.55-4.87) | 1.84 - 24.43 |
| **Guainia** | 83 | 0.04 | 2.24  (2.15-2.33) | 0.75- 6.66 | 16 | 0.05 | 2.19  (2.12-2.27) | 0.86- 6.66 |
| **Guajira** | 1,516 | 0.82 | 7.64  (7.34-7.95) | 2.57 - 22.68 | 201 | 0.64 | 4.44  (4.30-4.60) | 1.74 - 22.68 |
| **Guaviare** | 186 | 0.10 | 3.02  (2.90-3.14) | 1.01 - 8.96 | 17 | 0.05 | 2.26  (2.19-2.34) | 0.88 - 8.96 |
| **Huila** | 4,949 | 2.68 | 4.48  (4.31-4.66) | 1.51 - 13.30 | 839 | 2.70 | 5.08  (4.91-5.26) | 1.99 - 13.30 |
| **Magdalena** | 1,438 | 0.78 | 8.62  (8.28-8.97) | 2.90 - 25.59 | 194 | 0.62 | 4.53  (4.38-4.69) | 1.77 - 25.59 |
| **Meta** | 2,516 | 1.36 | 3.87  (3.71-4.03) | 1.30 - 11.47 | 287 | 0.92 | 3.06  (2.96-3.17) | 1.20 - 11.48 |
| **Nariño** | 4,182 | 2.27 | 8.23  (7.90-8.56) | 2.77- 24.42 | 531 | 1.71 | 6.63  (6.40-6.86) | 2.59 - 24.42 |
| **Norte de Santander** | 6,879 | 3.73 | 5.85  (5.63-6.08) | 1.97 - 17.38 | 1,428 | 4.60 | 6.47  (6.26-6.70) | 2.53 - 17.38 |
| **Putumayo** | 1,263 | 0.68 | 14.96  (14.37-15.57) | 5.04 - 44.40 | 110 | 0.35 | 8.90  (8.60-9.20) | 3.48 - 44.40 |
| **Quindio** | 1,865 | 1.01 | 3.38  (3.24-3.51) | 1.14 - 10.03 | 162 | 0.52 | 2.59  (2.51-2.68) | 1.01 - 10.03 |
| **Risaralda** | 2,812 | 1.52 | 4.94  (4.75-5.15) | 1.66 - 14.68 | 364 | 1.17 | 4.97  (4.81-5.14) | 1.95 - 14.68 |
| **San Andres** | 240 | 0.13 | 7.45  (7.16-7.75) | 2.51 - 22.13 | 37 | 0.12 | 4.62  (4.46-4.79) | 1.81 - 22.13 |
| **Santander** | 9,111 | 4.94 | 6.63  (6.37-6.89) | 2.23- 19.67 | 839 | 2.70 | 4.55  (4.40-4.71) | 1.78 - 19.67 |
| **Santa Marta** | 1,986 | 1.07 | 6.95  (6.69-7.24) | 2.34 - 20.65 | 334 | 1.07 | 5.31  (5.13-5.50) | 2.08 - 20.65 |
| **Sucre** | 2,311 | 1.25 | 7.88  (7.57-8.19) | 2.65 - 23.39 | 278 | 0.89 | 3.52  (3.40-3.64) | 1.38 - 23.39 |
| **Tolima** | 4,923 | 2.67 | 6.15  (5.92-6.40) | 2.07 - 18.27 | 510 | 1.64 | 4.62  (4.47-4.78) | 1.81 - 18.27 |
| **Valle** | 13,795 | 7.48 | 6.08  (5.84-6.33) | 2.05 - 18.07 | 2,113 | 6.80 | 6.00  (5.80-6.20) | 2.35 - 18.07 |
| **Vaupes** | 78 | 0.04 | 4.54  (4.36-4.72) | 1.53 - 13.48 | 14 | 0.04 | 2.21  (2.14-2.29) | 0.87 - 12.92 |
| **Vichada** | 72 | 0.039 | 5.57  (5.35-5.78) | 1.87 - 16.52 | 10 | 0.03 | 5.52  (5.34-5.71) | 2.16 - 16.52 |
| **Total** | 184340 |  |  |  | 31032 |  |  |  |

GW General Ward bed, ICU Intensive Care Unit, n number of hospital admissions entering each bed pathway , % proportion of hospital admissions entering each bed pathway, $x$ Median and 95% C.I., IQR Interquartile range.


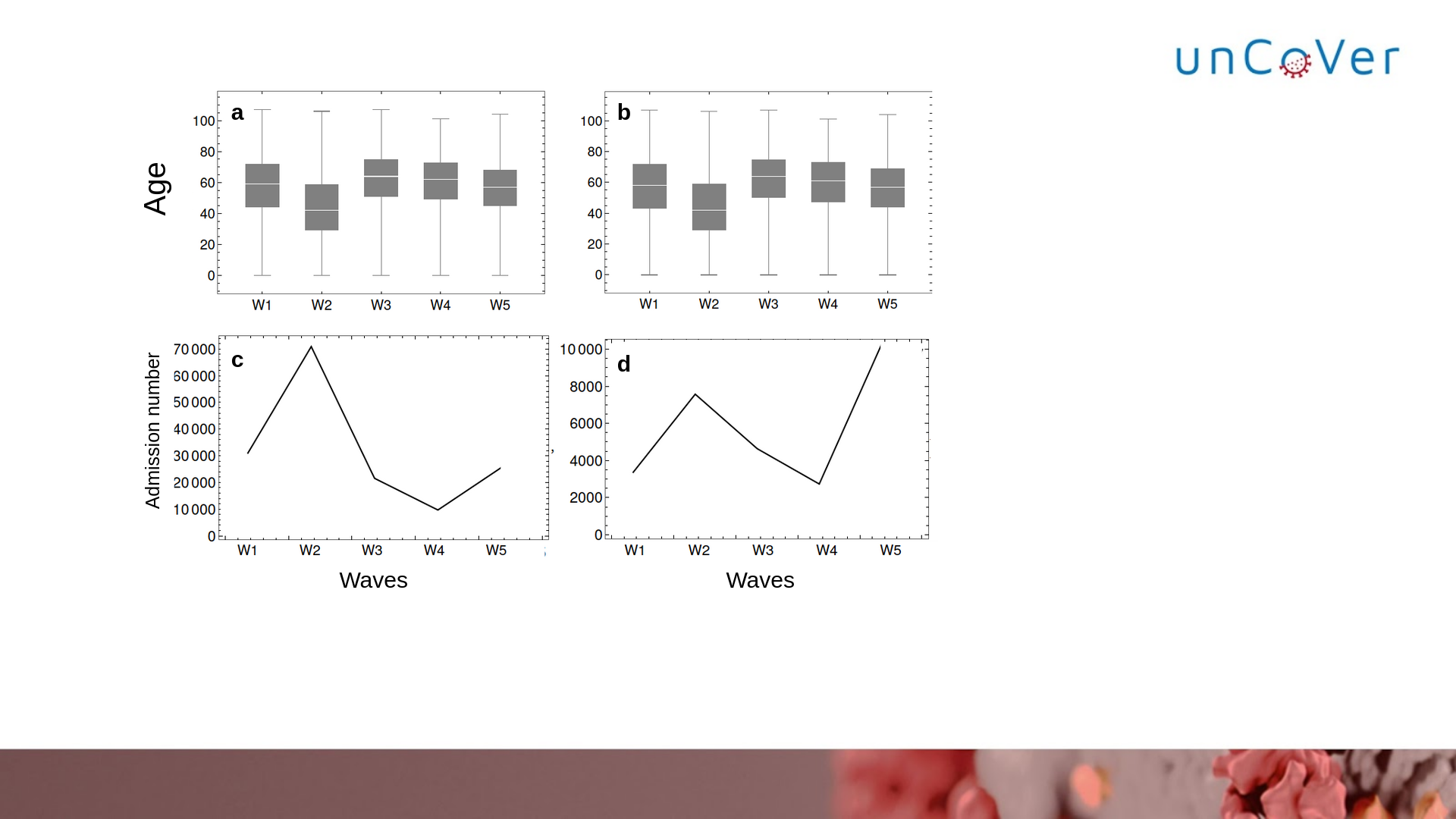


### S4 Fig. Age distribution and admission number throughout the waves. (a). Age distribution of patients with BP1 (only GW bed), (b). Age distribution of patients with BP2 (only ICU bed), (c). Admission number of patients with BP1 (only GW bed), (d). Admission number of patients with BP2 (only ICU bed).

**REFERENCES**

1. [Nesteruk I. COVID-19 Pandemic Dynamics: Mathematical Simulations. Springer Nature; 2021. 172 p.](http://paperpile.com/b/iYjjEL/CyLT)
